# Surface-enhanced Raman Spectral Measurements of 5-Fluorouracil in Saliva

**DOI:** 10.3390/molecules13102608

**Published:** 2008-10-22

**Authors:** Stuart Farquharson, Alan Gift, Chetan Shende, Frank Inscore, Beth Ordway, Carl Farquharson, John Murren

**Affiliations:** 1 Real-Time Analyzers, Inc., Middletown, CT 06457, USA; E-mails: Alan@rta.biz (A. G.), Chetan@rta.biz (C. S.), Inscore@rta.biz (F. I.); 2 Yale University, New Haven CT 06520, USA

**Keywords:** 5-FU, Chemotherapy, Drug analysis, Saliva analysis, Raman, SERS

## Abstract

The ability of surface-enhanced Raman spectroscopy (SERS) to measure 5-fluorouracil (5-FU) in saliva is presented. The approach is based on the capacity of Raman spectroscopy to provide a unique spectral signature for virtually every chemical, and the ability of SERS to provide μg/mL sensitivity. A simple sampling method, that employed 1-mm glass capillaries filled with silver-doped sol-gels, was developed to isolate 5-FU from potential interfering chemical components of saliva and simultaneously provide SERS-activity. The method involved treating a 1 mL saliva sample with 1 mL of acetic acid, drawing 10 μL of sample into a SERS-active capillary by syringe, and then measuring the SER spectrum. Quality SER spectra were obtained for samples containing as little as 2 μg of 5-FU in 1 mL saliva. The entire process, the acid pretreatment, extraction and spectral measurement, took less than 5 minutes. The SERS of 5-fluorouridine and 5-fluoro-2’-deoxyuridine, two major metabolites of 5-FU, were also measured and shown to have unique spectral peaks. These measurements suggest that disposable SERS-active capillaries could be used to measure 5-FU and metabolite concentrations in chemotherapy patient saliva, thereby providing metabolic data that would allow regulating dosage. Tentative vibrational mode assignments for 5-FU and its metabolites are also given.

## Introduction

5-Fluorouracil (5-FU) is often prescribed for solid tumors and colorectal carcinoma [1]. The success of 5-FU, like other chemotherapy drugs, is based on the higher replication rate of cancerous cells and corrupted biochemical synthesis, leading to cytosis. However, for most chemotherapy drugs these effects are not cancer cell specific, and normal cell growth throughout the body is also adversely affected. Furthermore, the body localizes or concentrates many of these drugs as they are processed by the liver and kidney resulting in damage to these organs. These dangerous side-effects preclude the use of traditional clinical trials involving numerous patients to establish statistical bases for dosages. Instead, initial dosage is based on the limited sets of previously treated patients, and the individual patient’s body surface area. Recently, the latter has been shown to be ineffective [2]. Determining safe and effective dosage has been, and remains, a significant challenge.

**Figure 1:**
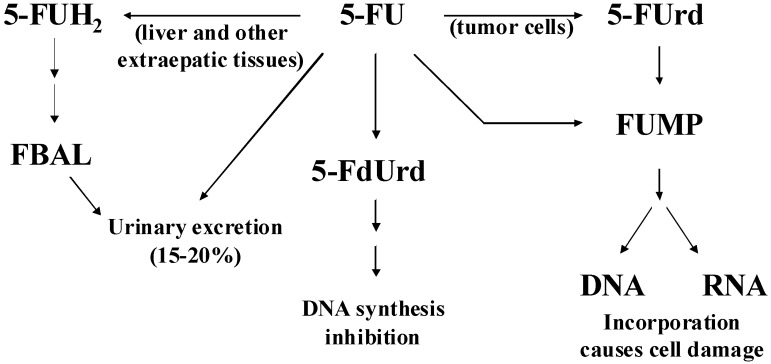
The metabolism pathways of 5-fluorouracil in humans (modification of Ref. [5]). Abbreviations: 5-FU, 5-fluorouracil; 5-FUH_2_, 5-fluoro-5,6-dihydrouracil; 5-FUrd, 5-fluorouridine; 5-FdUrd, 5-fluoro-2’-deoxyuridine; FUMP, 5-fluorouridine-5’-monophosphate; FBAL, α-fluoro-β-alanine.

The metabolism of 5-FU has been extensively studied and several well characterized biochemical pathways have been defined (Figure 1) [3,4,5]. In the anabolic pathway (cytotoxic mechanism) 5-FU is first converted to the nucleosides 5-fluorouridine (5-FUrd) and 5-fluoro-2’-deoxyuridine (5-FdUrd), and eventually to one of three active nucleotides: 5-fluorodeoxyuridine monophosphate (FdUMP), 5-fluorodeoxyuridine triphosphate (FdUTP), or 5-fluorouridine triphosphate (FUTP). FUTP and FdUTP are incorporated into RNA and DNA, respectively, impairing gene-encoding and ultimately preventing cell growth, while FdUMP inhibits the enzyme thymidine synthase and subsequently preventing DNA synthesis. In the catabolic pathway, dihydropyrimidine dehydrogenase (DPD) metabolizes 5-FU to an inactive form, 5-fluoro-dihydrouracil (5-FUH_2_). This is significant, in that there is a substantial genetic-based variation in the amount of DPD in individuals, and the amount of 5-FU metabolized can range from 15-80%. Consequently, employing a "standard" dose of 5-FU based on body surface area has led to severe toxicity, and even death, in individuals deficient in DPD [6,7].

A more direct method of regulating drug dosage is to monitor drug metabolism while it is being administered. Unfortunately, the current methods used to measure drug metabolism are severely limited. These analytical methods employ multiple steps to isolate the parent drug and its metabolites from the numerous biochemicals and biological components in blood serum and urine. For urine, drug isolation usually employs: 1) an organic solvent to isolate the drugs and metabolites from natural components, 2) chromatography to separate the drugs, metabolites, chemicals and biochemicals (typically gas or high performance liquid chromatography) [8,9], with detection and quantification by an ultraviolet detector (non-chemical specific), or more definitively by a fluorescent spectrometer [10,11] or mass spectrometer [12]. Inclusion of standards throughout this process is required to ensure measurement accuracy. Serum employs several additional steps, including centrifugation to remove red blood cells. These methods are labor intensive and time consuming (>hour). Furthermore, the large volumes of blood required (10 mL every 30 minutes) [13] could further jeopardize the patient’s health due to a depleted blood supply. Consequently, monitoring metabolism is limited to an as needed basis [14].

Saliva analysis has long been considered a potential alternative to the approaches described above, and recent research has shown that drug metabolism is often equally represented in saliva as it is in blood plasma, typically at μg/mL concentrations [15,16,17,18]. Analysis of saliva provides a highly desirable option, in that it is non-invasive, reduces the risk of HIV infection, is readily obtained, and is relatively easy to chemically analyze. Saliva is 99.5% water, and the concentrations of interfering physiological chemicals are typically 100 or more times less than in blood plasma or urine [19]. Current techniques for saliva analysis, however, like that of blood, require the use of 10 to 20 mL samples in order to enable chemical separation and detection of drugs and their metabolites, and such quantities are difficult for the patient to generate. A recent pharmacokinetic investigation has shown that 5-FU concentrations in saliva closely match those in plasma, reaching a maximum of 15-28 μg/mL and a three hour minimum of 0.1 μg/mL (patient dependent) [18].

One approach that has been used to measure chemicals at μg/mL concentrations is surface-enhanced Raman spectroscopy (SERS). SERS has the potential to perform this analysis with just a few drops of sample due to its extreme sensitivity demonstrated by single molecule detection [20,21]. In addition, the rich molecular vibrational information provided by Raman scattering yields exceptional selectivity and allows virtually any chemical to be identified, while also distinguishing multiple chemicals in mixtures [22,23]. Recently, SERS has been used to measure a wide range of different pharmaceuticals [24,25,26], as well as several chemotherapy drugs [27], including mitoxantrone [28,29,30], acridine drugs [31], and anthraquinone drugs [32].

5-FU has been given special attention in our work [33,34,35,36], since it is widely used, is well represented in saliva, and the metabolism of this drug varies enormously from individual-to-individual. Here we present the SERS analysis of 5-fluorouracil, 5-fluorouridine and 5-fluoro-2’-deoxyuridine. In the case of 5-FU, pH dependence and concentration studies are also provided, along with the development of a method to measure 2 μg in 1 mL of saliva. These measurements provide the foundation for developing simple sampling devices, such as disposable SERS-active capillaries, to measure chemotherapy drugs and their metabolites at physiological concentrations in less than a drop of saliva within 5 minutes. Such a device would allow monitoring drug metabolism and regulating dosage during administration, and ultimately improving cancer patient outcome on an individual basis.

## Results and Discussion

Surface-enhanced Raman spectra of chemicals are often different from their normal Raman spectral counterparts. This is largely due to surface interactions that enhance various vibrational modes to different extents resulting in spectral peaks shifts and changes in relative intensity. For this reason, the normal Raman spectrum of 5-FU, 5-FUrd, and 5-FdUrd were also measured to aid interpretation of the SERS spectra.

**Figure 2:**
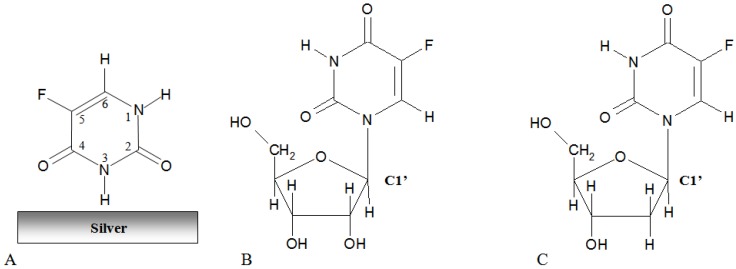
Chemical structure of A) 5-fluorouracil, B) 5-fluorouridine and C) 5-fluoro-2’-deoxyuridine. Each atom of the ring for 5-FU is labeled 1 to 6 to define substitution position.

**Figure 3:**
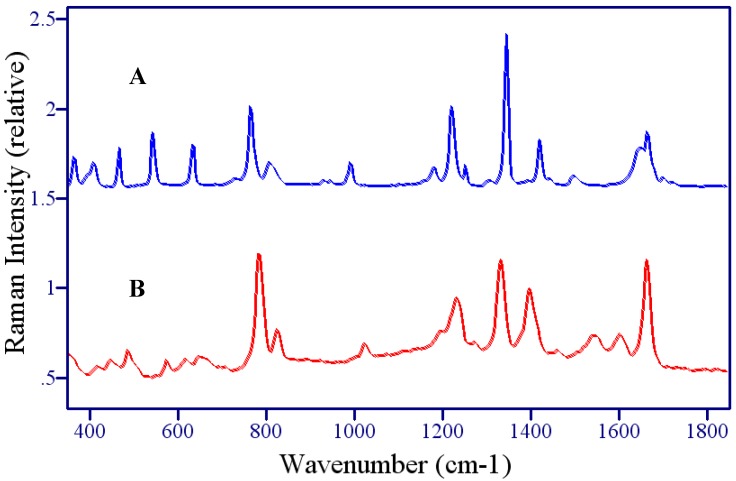
A) Raman and B) SER spectra of 5-fluorouracil. Conditions: A) crystalline powder, 150 mW of 785 nm, 1 min, B) 1 mg/mL, 100 mW of 785 nm, 2 min.

The molecular structure of 5-FU is similar to uracil except for a fluorine substitution at the C5 position on the ring (Figure 2). Consequently, most of the Raman peaks can be assigned to modes based on previous analysis of uracil [37,38]. Raman spectra of 5-FU have also been analyzed (Figure 3A), [39] recently with the aid of *ab initio* calculations [35,40], and the contributions of fluorine to vibrational modes have been added. Combining these assignments, the dominant peaks in the Raman spectrum of 5-FU at 365, 410, 467, 544, 635, 766, 1223, 1347, 1423, 1653 and 1669 cm^-1^ are assigned to one out-of-plane pyrimidine ring bending mode, four in-plane ring bending modes, the ring breathing mode, the ring plus C-F stretching mode, the ring plus C-H wagging mode, the ring plus the two N-H wagging modes, the C=C stretching mode, and the symmetric C=O stretching mode, respectively (Table 1). The addition of the fluorine atom, causes many peaks to shift, as expected, such as the ring breathing mode from 780 cm^-1^ for uracil to 766 cm^-1^ for 5-FU. This is consistent with theoretical calculations that predict a 32 cm^-1^ lower frequency shift for fluorine substitution. Albeit, the authors of a recent published Raman spectrum of 5-FU assign the 766 cm^-1^ peak to the out-of-plane C4=O carbonyl bend [40].

The surface-enhanced Raman spectrum of 5-FU is dominated by peaks at 786, 1234, 1334, 1400, and 1667 cm^-1^ (Figure 3B), and, based on peaks at similar frequency in the Raman, are assigned to the pyrimidine ring breathing mode, the trigonal ring plus C-F stretching mode, a ring plus C-H wagging mode, a ring plus N-H wagging modes, and the symmetric C=O stretching mode (Table 1). Several peaks also appear in the low frequency region (400-650 cm^-1^), which are assigned to ring-bending modes as a group. The shift in frequency compared to the Raman spectrum for most of these modes suggests strong interaction with the silver surface, which enhance various vibrational modes to different extents resulting in spectral peak shifts and changes in relative intensity. Three previous studies showed shifts of very similar amounts for corresponding modes in uracil [40,41,42,43], and in one case it was concluded that the interaction was through a deprotonated nitrogen [43], while another study, concluded that the interaction was through the N3 position (see below) [35]. The direction of the shifts also suggest an increase in bond strength for the ring breathing mode and the ring plus C-F stretching mode (766 to 786 cm^-1^ and 1223 to 1234 cm^-1^, respectively), and a decrease in bond strength for the ring plus C-H and N-H wagging modes (1347 to 1334 cm^-1^ and 1423 to 1400 cm^-1^, respectively).

In addition to these shifts in frequency, several peaks change relative intensity in the SERS spectrum compared to the Raman spectrum. In particular, the 786 cm^-1^ ring breathing mode and the 1667 cm^-1^ C=O stretching mode gain intensity, the 1653 cm^-1^ C=C stretch disappears, and new peaks appear at 1545 and 1606 cm^-1^. Together these changes are consistent with previous assertions for uracil, which state that the molecule is oriented to the surface end-on, based on the fact that modes perpendicular to the surface are most enhanced [43]. In this orientation the ring breathing mode is perpendicular to the surface, along with the trigonal ring breathing mode that now appears at 1606 cm^−1^. The same trigonal mode has been observed in the SER spectra of pyrazine at 1590 cm^-1^ [44], supporting this assignment. Furthermore, the C=C stretch at 1565 cm^-1^ disappears, which would occur if this mode were parallel to the surface as in the case of an end-on orientation to the surface through N3. The disappearance of this mode in the SERS spectrum has also been reported for 6-aminouracil [42]. The low frequency peaks (400-650 cm^-1^) all lose intensity as might be expected for ring bending modes, which vibrate more parallel than perpendicular to the surface.

It is clear from Figure 1, that it is highly desirable to not only measure 5-FU, but also 5-FUH_2_, FBAL, 5-FUrd, 5-FdUrd, and FUMP and show that their SER spectra are different so that concentrations can be determined. Furthermore, if these chemicals can be measured in the same saliva sample, the need to determine absolute concentrations will be minimized, since relative concentrations can be used to determine pharmacokinetics. Unfortunately, only 5-FUrd and 5-FdUrd were available at the time of this study. Since the concentration of 5-FUH_2_ in saliva has added importance in that its ratio to 5-FU would indicate a patient’s DPD level, it is worth noting that the SER spectra of the analogs uracil and dihydrouracil are substantially different. In particular, the pyrimidine ring breathing mode at 805 cm^-1^ for uracil shifts to 725 cm^-1^ for dihydrouracil [45].

**Figure 4:**
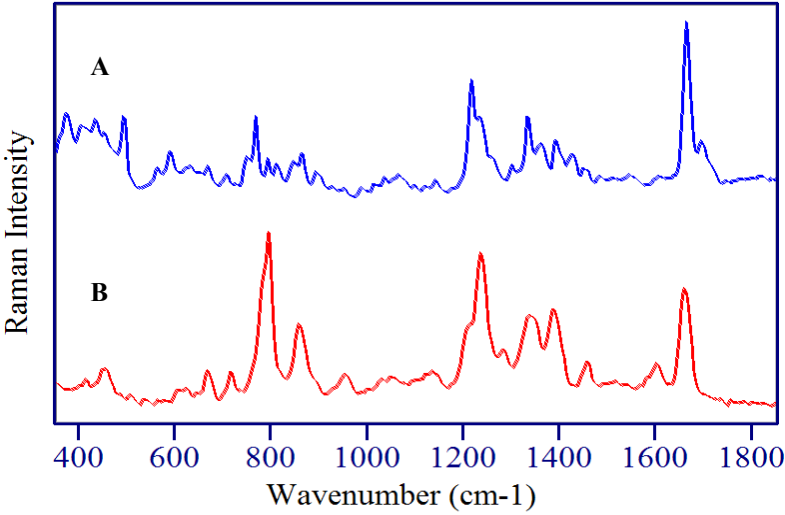
A) Raman and B) SERS spectra of 5-fluorouridine. Conditions: A) crystalline powder, 100 mW of 785 nm, 5 min, B) 1 mg/mL, 100 mW of 785 nm, 3 min.

**Figure 5:**
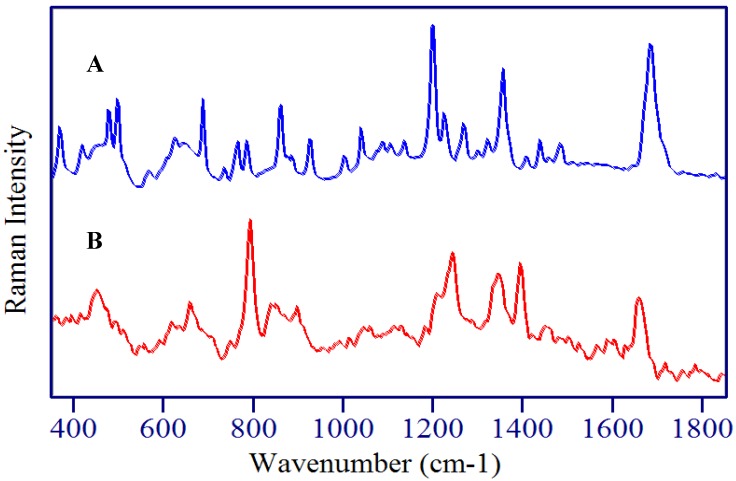
A) Raman and B) SER spectra of 5-fluoro-2’-deoxyruidine. Conditions: A) crystalline powder, 100 mW of 785 nm, 5 min, B) 1 mg/mL, 100 mW of 785 nm, 3 min.

5-Fluorouridine and 5-fluoro-2’-deoxyuridine differ in structure by the addition of D-ribose and D-deoxyribose moieties at the N1 position of the uracil ring, respectively (Figure 2). Although the chemical structures for these two metabolites are extremely similar, the Raman spectra of the crystalline powders are surprisingly different (Figure 4 and Figure 5), presumably due to a combination of intra- and inter-molecular forces. For the former, rotation about N1 and C1’, and puckering of the ribose moiety can influence vibrations, and for the latter, hydrogen bonding and the local crystal environment can influence vibrations [46].

A comparison of the normal Raman and SERS spectra for 5-FUrd, crystalline powder and 1 mg/mL solution, respectively, is shown in Figure 4. The frequencies of the peaks are listed in Table 1. The normal Raman spectrum has many of the same features as 5-FU with peaks at 767, 1233, 1334, 1664 and 1695 cm^-1^ corresponding to the pyrimidine ring breathing mode, the ring plus C-F stretch, the ring plus C-H wag, the C=C stretch, and the symmetric combination of the two C=O stretches, respectively. The intensity of the normal Raman spectrum is significantly less in comparison to the 5-FU spectrum, especially for the ring modes. This is due mainly to the reduction in symmetry of the molecule with the addition of the ribose. Other peaks in the Raman spectrum appear at 492 cm^-1^, a group between 800 to 900 cm^-1^, and an intense peak at 1216 cm^-1^. These peaks are all assigned to combination modes of the uracil ring plus ribose vibrations.

The SERS spectrum of 5-FUrd is dominated by peaks at 794, 859, 1236, 1339, 1388 and 1661 cm^-1^, which are assigned to the pyrimidine ring breathing mode, the trigonal ring plus C-F stretch, the ring plus C-F stretch, the ring plus C-H wag, the ring plus N-H wag, and the C=O symmetric stretch, respectively. As with 5-FU, the ring containing vibrational modes are enhanced the most indicating a surface adsorption interaction through the N3 with an upright geometry. Furthermore, the ribose modes do not appear to be enhanced, suggesting that this part of the molecule is directed away from the surface, which again supports an N3 surface interaction.

A comparison of the normal Raman and SERS spectra for 5-FdUrd, crystalline powder and 1 mg/mL solution, respectively, are shown in Figure 5. Again, the frequencies of the peaks are listed in Table 1. The uracil ring vibrations in the normal Raman spectrum have lost intensity with respect to 5-FU, due to the reduction in symmetry, while the ribose containing modes have gained intensity compared to 5-FUrd. The peaks associated with the uracil ring at 764, 1225, 1355, 1685 cm^-1^ and the shoulder at 1713 cm^-1^ are assigned to the pyrimidine ring breathing mode, the ring plus C-F stretch, the ring plus C-H wag, the C=C stretch, and the symmetric C=O stretches, respectively. Other intense peaks at 496, 687, 860, 925, and 1199 cm^-1^ are all associated with combination modes containing both uracil ring and ribose vibrations.

**Table 1:** Tentative vibrational mode assignments for the normal Raman (NR) and SERS peaks for 5-fluorouracil (5-FU) and its metabolites 5-fluorouridine (5-FUrd), and 5-fluoro 2’deoxyuridine (5-FdUrd).

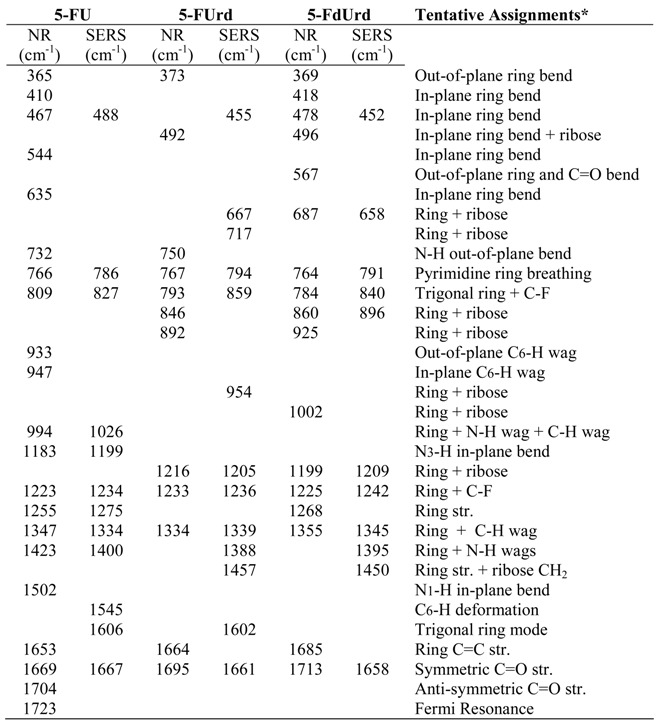

*Vibrational mode assignments are based on references [36,37,38,39,40,44], and this work.

The SERS spectrum of 5-FdUrd is again dominated by the uracil ring vibrational modes. The peaks at 791, 840, 1242, 1345, 1395, and 1658 cm^-1^ are assigned to the pyrimidine ring breathing mode, the trigonal ring plus C-F stretch, the ring plus C-F stretch, the ring plus C-H wag, the ring plus N-H wag, and the symmetric C=O stretches, respectively. There is also an additional feature at 896 cm^-1^ that represents a combination mode containing ring and ribose vibrational character. The appearance of this mode may be due to a different intramolecular orientation between the uracil and ribose moieties compared to 5-FUrd. In spite of the increased ribose features in the SERS spectrum, the uracil ring mode still dominates, and clearly establishes that this metabolite also interacts through the N3 in an end-on geometry. 

**Figure 6:**
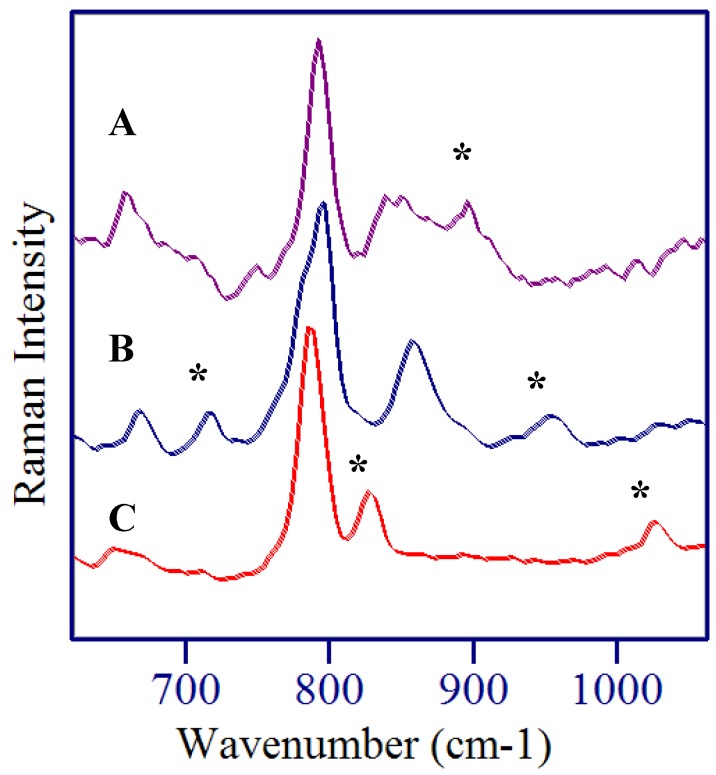
SERS spectra of A) 5-fluoro-2’-deoxyruidine, B) 5-fluorouridine, and C) 5-fluorouracil. Unique peaks identified by *. Conditions as in Figure 3, Figure 4 and Figure 5.

As stated previously, the SERS spectra for these three species are similar with many of the same vibrational modes being enhanced. Nevertheless, there are unique spectral features that can be used to identify each component. For example, Figure 6 shows that the 827 and 1026 cm^-1^ peaks are unique to 5-FU, the 716 and 954 cm^-1^ peaks are unique to 5-FUrd, and the 896 cm^-1^ peak is unique to 5-FdUrd. It therefore seems likely that simple spectral analysis could be used to identify and quantify each of these three species in a mixture. In the event that this approach is insufficient, chemometric-based software programs are available, which use the entire spectrum to identify and quantify multiple components in mixtures.

**Figure 7:**
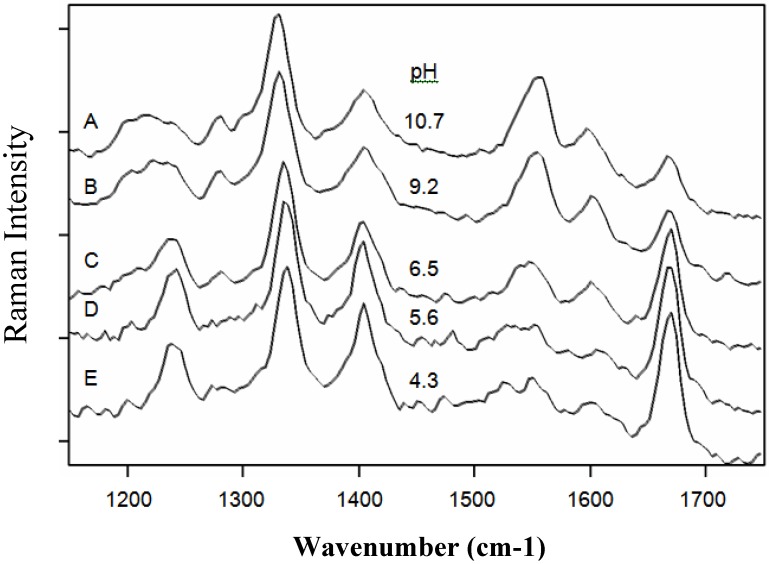
A) Surface-enhanced Raman spectra of 1 mg/mL 5-FU in water at pH A) 10.7, B) 9.2, C) 6.5, D) 5.6, and E) 4.3. Conditions: 100 mW of 785 nm, 2 min.

5-FU is a weak acid with a pKa of 7.93 [47], and consequently, depending on the ionic form, the pH can also strongly influence the molecule to surface interaction [48]. The pH dependence of the SERS spectrum is essential if quantitative measurements are to be made. Figure 7 shows the SERS spectra of 5-FU for selected pH values of 4.3, 5.6, 6.5, 9.2, and 10.7. The peaks at 1234, 1400 and 1667 cm^-1^ decrease in intensity, while the peaks at 786 and 1335 cm^-1^ remain constant as the pH is made basic. Yet, the latter two peaks both shift in frequency towards each other by some 8 cm^-1^ as the pH is changed from 3.3 to 10.7. Although, this shift appears to trend the pH, it is equivalent to the spectral resolution of the measurements and was not further analyzed. In addition, two new peaks appear at 1545 and 1606 cm^-1^. The decrease in the intensities of the 1234, 1400 and 1667 cm^-1^ peaks are a consequence of the anion being formed. Deprotonation occurs at the N3 position [47], which essentially removes this interaction with the chloride anions on the silver surface, as well as that of the nearby carbonyl groups. It also appears that the intensity of the 1400 cm^-1^ ring plus N-H wagging mode is reduced by approximately 50%, consistent with deprotonation and elimination of one of the two modes. The appearance of the peak at 1545 cm^-1^, assigned to the C6-H deformation mode, suggests that this part of the molecule now interacts with the chloride anion coated surface. The 1606 cm^-1^ peak is tentatively assigned to a trigonal ring mode, based on the same mode observed in the SERS spectra of pyrazine at 1590 cm^-1^[44], and the fact that formation of the anion leads to a high degree of π-electron delocalization [47].

**Figure 8:**
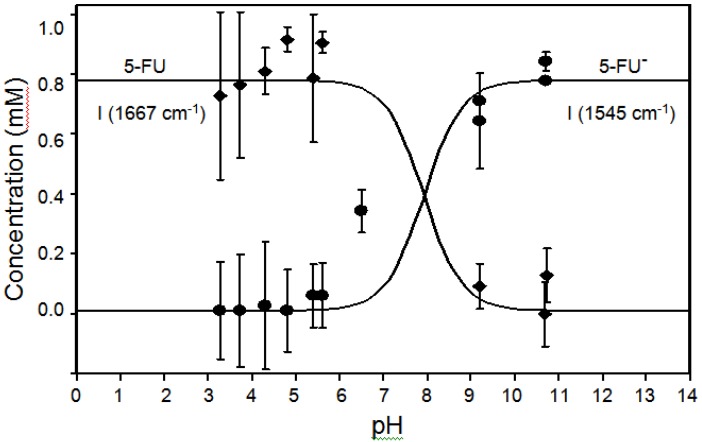
Plot of the 1545 (●) and 1667 cm^-1^ (♦) peak intensities (heights normalized to the 1335 cm^-1^ peak height) as a function of pH representing 5-FU^-^ and 5-FU, respectively. Lines represent predicted relative concentrations based on the pKa of 7.93.

The intensities of the 1545 and 1667 cm^-1^ peaks, normalized to the 1335 cm^-1^ peak intensity, are plotted in Figure 8 as a function of pH and compared to the calculated concentration of the neutral and anionic forms based on the pKa.

**Figure 9:**
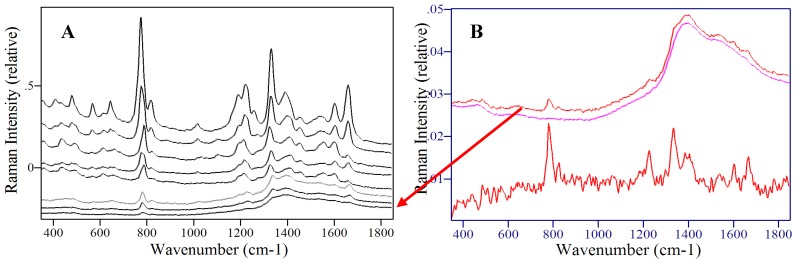
SER spectra of 5-FU at A) 500, 100, 10, 5, 1, 0.5, 0.1, 0.01 μg/mL (top to bottom) and B) 10 ng/mL 5-FU before (top) and after (bottom) subtraction of glass capillary background spectrum (middle). Conditions: silver-doped sol-gels in capillaries, 100 mW of 785 nm, 3-min.

**Figure 10:**
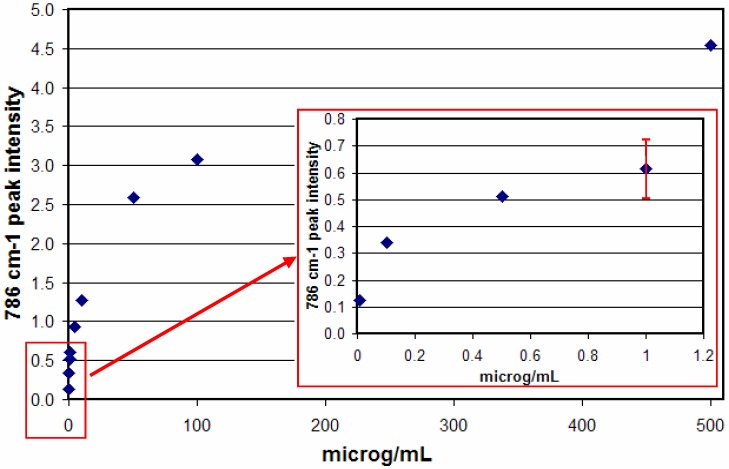
SER spectral response as a function of 5-FU concentration (0.01 μg/mL is lowest point). Conditions: 100 mW of 785 nm, 3 min. Error is ~ 20% RSD, 18% shown for 1 μg/mL.

The decrease in the latter peak and increase in the former peak coincident with the pKa supports their respective assignment to the neutral and anionic forms. It is important to note that the 786 and 1335 cm^-1^ peak intensities are nearly constant as a function of pH and can therefore be used for quantitative analysis. Next, the ability of SERS to detect 5-FU at relevant concentrations was examined by measuring samples at decreasing concentrations. Specifically, samples of 500, 100, 10, 5, 1, 0.5, 0.1, and 0.01 μg/mL were prepared and measured in the SERS-active capillaries (Figure 9A). At the lowest concentrations, a luminescent background becomes prominent, which is due to the metal impurities in the glass capillaries. This can be subtracted to reveal the 5-FU peaks (Figure 9B). The SERS intensity, measured as the 785 cm^-1^ peak area, as a function of concentration follows a Langmuir-Blodgett response, since it is a function of available silver surface area (Figure 10). 

**Figure 11:**
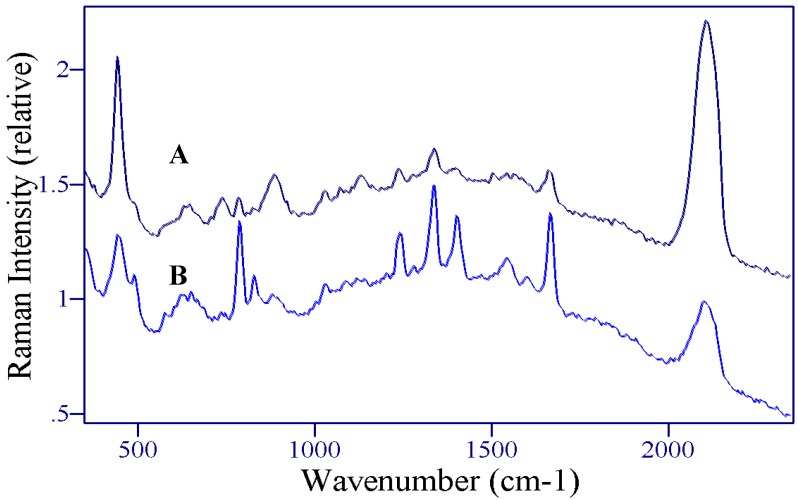
Surface-enhanced Raman spectra of 5FU in A) saliva and B) 1:3 saliva:water. Conditions: 1 mg/mL, 100 mW of 785 nm, 1 min. Same initial saliva sample and intensity scale.

**Figure 12:**
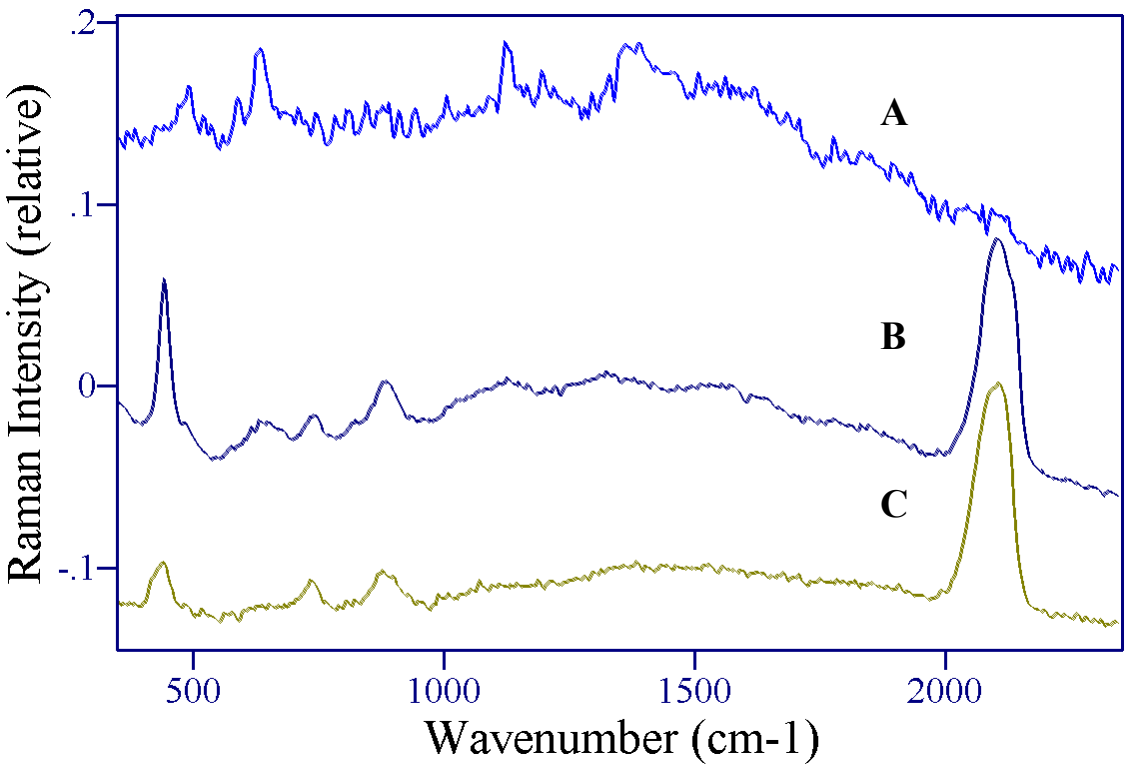
SER spectra of A) saliva-1, B) saliva-2, and C) KSCN. Conditions: 1 mg/mL, 100 mW of 785 nm, 1 min. Note the top spectrum was multiplied by 10 so features could be observed.

The first sample of 5-FU in saliva employed a relatively high concentration at 1 mg/1 mL. The 5-FU was artificially added to saliva and then drawn into SERS-active capillaries and measured. Although the 5-FU peaks are discernable, the spectrum is dominated by two additional peaks at 445 and 2095 cm^-1^ (Figure 11A). These peak frequencies match those previously reported for thiocyanate measured on a silver electrode [49]. This assignment was confirmed by preparing a 1 mg/mL thiocyanate sample and comparing it to the original saliva sample without 5-FU (Figure 12). Thiocyanate is physiologically extracted from some vegetables (e.g. broccoli) and appears in the saliva as an antibacterial agent [50]. However, after measuring several additional saliva samples, it was found that thiocyanate is more often NOT observed in saliva as shown in Figure 12A. Although thiocyanate does not spectrally interfere with 5-FU, nor is it likely to interfere with spectra of the 5-FU metabolites or other drugs, it or other anions in saliva, such as phosphates, nitrates or sulfates, could block the SERS-generating metal surfaces. Consequently methods were investigated to remove such anions.

**Figure 13:**
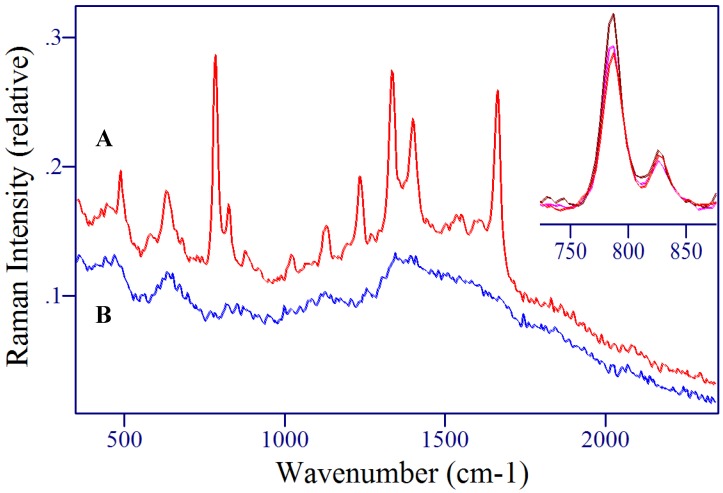
SER spectra of 5-FU in pH adjusted saliva for A) 50 μg/mL 5-FU and B) un-doped saliva. Inset: 3 repeat measurements. Conditions: 100 mW of 785 nm, 1 min.

**Figure 14:**
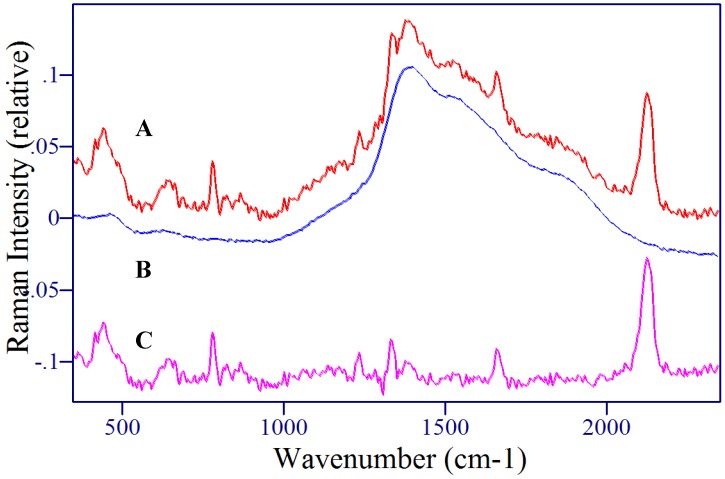
2 μg/mL 5-FU A) before and C) after subtraction of B) the glass capillary background. Conditions: HCl washed silver-doped sol-gel capillaries, 100 mW of 785 nm, 1min.

Fortuitously, dilution of the sample with water by a factor of two, intended to solvate 5-FU, but not the large biochemicals of saliva, such as α-amylase and mucin, also substantially reduced the thiocyanate signal intensity (Figure 11B). This prompted investigation of pH as a means to remove anions from the sample solution or exclude them from the silver surface. First, sodium hydroxide was added to the sample so that NaSCN would form and precipitate out of solution. Although this may have worked, a quality SER spectrum of 5-FU was not obtained. Second, the charge on the electropositive silver surface was made negative by treatment with HCl, which produced a Cl^-^ layer to repel anions. This also required acidifying the sample solution so that the 5-FU present would be predominantly neutral and attracted to the silver surface to generate SERS (see Figure 9). Acetic acid produced the desired effect. Figure 13 and Figure 14 shows the results for two different saliva samples to which 50 and 2 μg/ml 5-FU were added, respectively. In each case, 1 mL acetic acid was added to a 1 mL sample, stirred for 2 minutes, approximately 10 μL were drawn into SERS-active capillaries by syringe, that were first treated with 5 mM HCl, and a 1 minute SER spectrum was measured.

The 50 μg/mL 5-FU sample produced a quality spectrum (Figure 13, signal-to-noise ratio equaled 72 for the 786 cm^-1^ peak) with good reproducibility, as shown for three median spectra (Figure 13 inset). A SERS measurement of the same saliva without 5-FU produces a background spectrum, devoid of thiocyanate peaks (Figure 13B). The spectral quality of the 2 μg/mL 5-FU sample was considerably lower, as the spectrum was dominated by the glass capillary luminescence (Figure 14). Nevertheless, subtraction of this background reveals the characteristic 5-FU spectrum, which in this case includes thiocyanate peaks (Figure 14C). The 785 cm^-1^ peak height for this ~1 μg/mL saliva plus acid sample is approximately the same as the 0.01 μg /mL sample in water. This suggests that as little as 1/100^th^ of the 5-FU reaches the silver surface and gets spectrally enhanced. It further suggests that, at least at this concentration, thiocyanate, other ions, proteins or enzymes block the silver surface and/or sol-gel pores, reducing sensitivity. Nevertheless, the measured concentration is equivalent to that required to evaluate dosage, and only a 10 μL sample (1/5^th^ of a drop) was needed. Furthermore, the entire measurement, including acid pretreatment was accomplished in 4 minutes.

## Conclusions

In this article the surface-enhanced Raman spectral measurements for 5-fluorouracil, 5-fluorouridine and 5-fluoro-2’-deoxyuridine are described and tentative vibrational assignments are given. The SER spectra for these drugs were sufficiently different that the parent drug and its metabolites could be uniquely identified by this spectroscopy. Glass capillaries filled with silver-doped sol-gels were used to perform these measurements, as well as those of 5-FU artificially added to saliva. A reasonable signal-to-noise ratio spectrum was obtained for 2 μg of 5-FU in 1 mL saliva, consistent with the expected physiological range of 1-30 μg/mL for this chemotherapy drug. The measurement, including pretreatment with acetic acid and the spectral acquisition, was accomplished in less than 4 minutes. The ability to measure 5-FU at μg/mL concentrations in less than 5 minutes presented here brings us closer to our goal of developing disposable SERS-active capillaries or lab-on-chip devices that could be used to measure 5-FU and metabolite concentrations in chemotherapy patient saliva and thereby provide metabolic data that would allow regulating dosage on a patient-by-patient basis.

## Experimental

### General

5-Flourouracil (2,4-dihydroxy-5-fluoropyrimidine), 5-fluorouridine, 5-fluoro-2’-deoxyuridine, and leucovorin (N5-formyl-5,6,7,8-tetrahydropteroyl-L-glutamic acid), and all chemicals used to prepare the silver-doped sol-gel coated vials and capillaries were obtained from Sigma-Aldrich (St. Louis, MO) and used without further purification.

### Solutions and measurements

5-FU concentrations from 0.01 to 1,000 μg/mL were prepared in HPLC grade water and 2 to 1,000 μg/mL in saliva. The saliva used was donated by employees of Real-Time Analyzers, and the pH was measured at 6.95 to 7.05, but the exact composition was not determined. For the pH study of 5-FU, a 1 mg/mL stock solution was prepared, adjusted to pH 3.7 using 0.1 M HNO_3_, and then added to a 2 mL SERS-active vial (Real-Time Analyzers, *Simple SERS Sample Vials*, Middletown, CT). After the SERS measurement, the 2 mL solution was returned to the stock solution and made basic using 0.1 M KOH. Prior to re-addition to the same SERS-active vial, the vial was first rinsed three times with distilled water, then twice with the new solution prior to SERS measurement. This procedure was repeated until a pH of 10.7 was obtained and measured. Then the entire sequence was repeated, but instead 0.1 M HNO_3_ was used to make the solution incrementally acidic to a final measurement pH of 3.3. In all, pH measurements were performed at 3.3, 3.7, 4.3, 4.8, 5.4, 5.6, 6.5, 9.2, and 10.7.

The remaining measurements were performed in SERS-active capillaries. SER-active vials and SER-active capillaries were prepared according to published procedures using a silver precursor and an alkoxide precursor [51,52,53,54]. The alkoxide precursors were mixed with silver amine precursor in an 8/1 v/v ratio. In the case of the vials, the silver amine precursor consisted of a 5/1 v/v ratio of 1N AgNO_3_ to 28% NH_3_OH, while the alkoxide precursor consisted of a 2/1 v/v ratio of methanol to tetramethyl orthosilicate. Then 140 μL were introduced into 2 mL glass vials, which were then spin-coated. In the case of the capillaries, the alkoxide consisted of a 5/1/1 v/v/v ratio of methyltrimethoxysilane, tetramethyl orthosilicate, and octadecyltrimethoxysilane, and 15 μL were drawn by syringe into 1-mm diameter glass capillary to coat an ~15 mm length. In both cases, after sol-gel formation, the incorporated silver ions were reduced with dilute sodium borohydride. However, in the case of the capillaries, they were also rinsed with a 5 mM HCl solution to provide a negative charged layer and improve the SERS-activity. It has been shown that a layer of negative charge on silver provided by chloride anions can greatly enhance the interaction of some analytes and substantially increase surface-enhanced Raman scattering [55,56]. In fact the SERS of uracil in 0.1M KCl obtained on a silver electrode was found to be substantially more intense than uracil obtained on silver embedded in a sol-gel [57].

The sample solutions were either added by pipette into the SERS-active vials or drawn into SER-active capillaries using a syringe. In both cases the vial or capillaries were mounted horizontally on an XY positioning stage (Conix Research, Springfield, OR) such that the focal point of an f/0.7 aspheric lens was positioned just inside the glass wall. Further details of the apparatus can be found in [36]. The lens focused the beam into the sample and collected the scattered radiation back along the same axis. A dichroic filter (Omega Optical, Brattleborough, VT) was used to reflect the excitation laser to the lens and pass the Raman scattered radiation collected by the lens. An f/2 achromat was used to collimate the laser beam exiting a 200 μm core diameter source fiber optic, while a second f/2 achromat was used to focus the scattered radiation into a 365 μm fiber optic (Spectran, Avon, CT). A short pass filter was placed in the excitation beam path to block the silicon Raman scattering generated in the source fiber from reflecting off sampling optics and reaching the detector. A long pass filter was placed in the collection beam path to block the sample Rayleigh scattering from reaching the detector.

An FT-Raman spectrometer (Real-Time Analyzers, model IRA-785, Middletown, CT) equipped with a 785 nm diode laser and a silicon photo-avalanche detector was used to acquire the surface-enhanced and normal Raman spectra. The spectrometer was used to deliver 50 to 300 mW of power at the SERS and Raman samples and generate spectra with 8 cm^-1^ resolution. Since these capillaries are in the developmental stage, and some non-uniformity in sol-gel structure still exists, each capillary was measured at 9 positions along the length of the capillary with 1-mm spacing, then the three high and three low peak intensity values were discarded and the three middle values averaged to yield the reported spectra. All peak intensities used for calculations were measured peak heights.
